# Aspergillus keratitis presenting with an immune ring: a case report and literature review

**DOI:** 10.1186/s12348-026-00600-2

**Published:** 2026-06-05

**Authors:** Mehrnaz Atighehchian, Mehran Zarei-Ghanavati, Zohreh Abedinifar, Marzieh Shizari

**Affiliations:** 1 Farabi Eye Hospital, Tehran University of Medical Sciences, Tehran, Iran; 2 Noor Ophthalmology Research Center, Noor Eye Hospital, Tehran, Iran; 3 Microbiology Unit, Department of Pathology, Farabi Eye Hospital, Tehran University of Medical Sciences, Tehran, Iran

**Keywords:** Fungal keratitis, Immune-like ring, *Aspergillus*

## Abstract

**Purpose:**

To describe the clinical presentation, diagnostic challenges, and management of fungal keratitis (FK) caused by *Aspergillus* spp. presenting with a delayed immune-like ring infiltrate.

**Methods:**

We report a case describing the clinical course, diagnostic workup, and treatment of a patient with FK initially misdiagnosed as herpes simplex keratitis.

**Results:**

A 68-year-old woman presented with a three-week history of decreased vision and ocular pain in the right eye. She had previously been treated with topical chloramphenicol 0.5%, frequent topical betamethasone, and oral acyclovir for presumed herpes simplex keratitis. Despite two weeks of treatment, the corneal findings progressed. At presentation to our center, a yellow-white stromal infiltrate with a surrounding immune-like ring in the inferonasal periphery was observed, along with stromal thinning and signs of impending perforation. Corneal scraping confirmed *Aspergillus* spp. Topical betamethasone was discontinued, and topical voriconazole 1% with chlorhexidine 0.2% was initiated. Cyanoacrylate glue was applied to manage stromal melting. Following antifungal therapy and withdrawal of topical corticosteroids, the condition stabilized.

**Conclusion:**

Immune-like ring formation may be observed in FK due to immune-mediated mechanisms. Despite this immune component, topical corticosteroids should not be used empirically, as they may exacerbate fungal infection and worsen clinical outcomes. It is essential to perform corneal sampling and microbiological confirmation before initiating steroids in cases of immune-like ring lesions. This helps rule out infectious causes, especially FK.

## Introduction

Corneal ring infiltrates are ring-shaped stromal opacities, which can occur in both infectious and noninfectious keratitis [1]. They can be classified as infective, with viable microorganisms present, or sterile, resulting from an immune-mediated type III hypersensitivity reaction. The sterile variant, known as a Wessely ring, is caused by the deposition of antigen-antibody complexes [1, 2].

In fungal keratitis (FK), immune ring formation is uncommon and is thought to result from a host immune response to fungal antigens rather than direct fungal invasion [3]. It typically develops gradually, appearing 10 to 14 days after disease onset, and is characterized by a clear zone separating the ring from the primary corneal lesion [1, 4]. Accurate diagnosis requires a high index of suspicion, as FK with an immune-like ring infiltrate may be mistaken for herpes simplex keratitis or Acanthamoeba keratitis (AK) [5, 6]. This led to inappropriate prescriptions of topical corticosteroids, potentially worsening the infiltration [5]. Microbiological confirmation is vital, as it supports early diagnosis, guides appropriate antifungal therapy, and prevents severe complications [1].

Fungal keratitis is less commonly associated with immune rings, and *Aspergillus* spp. represent a relatively uncommon fungal pathogen, which emphasizes the unusual and distinctive nature of this case [3]. This case is distinguished by the uncommon combination of Aspergillus keratitis presenting with a delayed immune-like ring infiltrate, initially misdiagnosed as herpetic keratitis and modified by prior corticosteroid therapy and highlights a diagnostic pitfall and underscores the importance of early microbiological confirmation in atypical ring infiltrates.

## Case report

A 68-year-old woman presented with a three-week history of decreased vision and ocular pain in her right eye. Her medical history was significant for uncontrolled diabetes mellitus. She also had a unilateral pre-existing corneal scar of unclear etiology, which may have increased her susceptibility to fungal infection. There was no history of chronic ocular surface disease, recent ocular trauma, ocular surgery, and contact lens wear. She reported a history of a corneal scar from several years prior; the exact etiology was unclear. Best-corrected visual acuity (BCVA) was recorded at 1.0 LogMAR. She had been previously treated at another center with topical chloramphenicol 0.5%, frequent topical betamethasone, and oral acyclovir 400 mg twice daily for herpes simplex keratitis. Despite two weeks of treatment, her symptoms worsened.

**Fig. 1:**
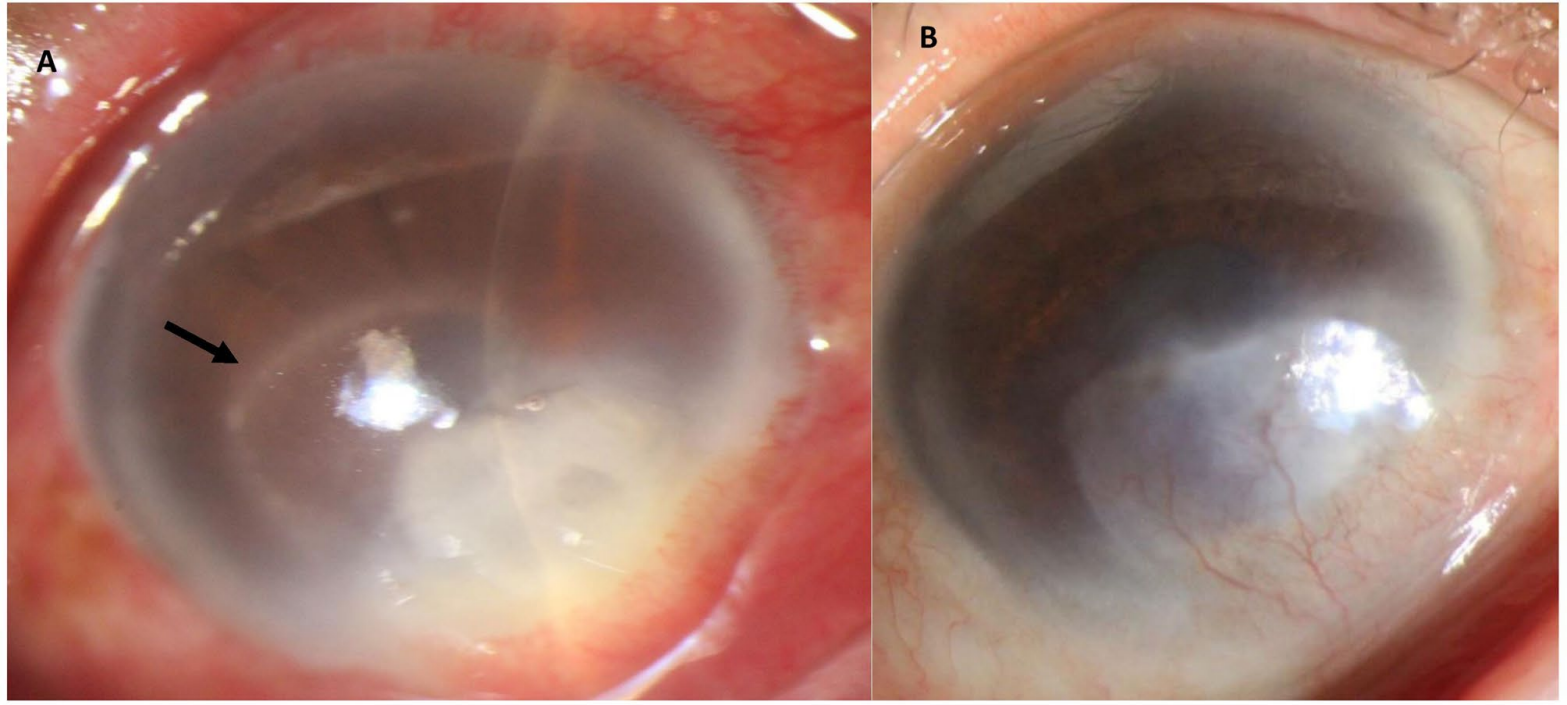
Clinical presentations. (**A**) Slit-lamp biomicroscopy shows a well-demarcated 5 × 4 mm peripheral stromal infiltrate extending to the limbus with central stromal thinning. A narrow, yellowish, incomplete ring-shaped infiltrate with a surrounding clear zone between the cornea-limbal infiltration is also visible (arrow). (**B**) Scar formation with superficial vascularization after three months

Slit-lamp biomicroscopy at presentation demonstrated a well-demarcated yellow-white stromal infiltrate measuring 5 × 4 mm in the inferonasal peripheral cornea, extending to the limbus and resulting in localized limbitis. Central stromal thinning measuring 3 × 3 mm and involving more than 50% of the stromal depth was noted, conferring a high risk of perforation. In addition, a narrow, yellowish, incomplete immune-like ring infiltrate was noted, separated from the primary lesion by a clear zone and more prominent toward the advancing edge of the ulcer. (Fig. 1A). Examination of the left eye was unremarkable, with no evidence of active or prior ocular disease. The posterior segment showed no involvement of the vitreous or retina. Upon admission, corneal scraping was performed under topical anesthesia using preservative-free proparacaine 0.5%. After gentle debridement of necrotic epithelium, samples were obtained using a sterile 15-blade. Sequential scrapings were taken from both the base and advancing edge of the ulcer. Multiple samples were collected for microbiological investigations, including 10% potassium hydroxide (KOH) wet mount preparation, Gram staining, and culture on blood agar, chocolate agar, and Sabouraud dextrose agar, as well as polymerase chain reaction (PCR) for viral analysis. All samples were processed promptly to maximize microbiological yield.

**Fig. 2:**
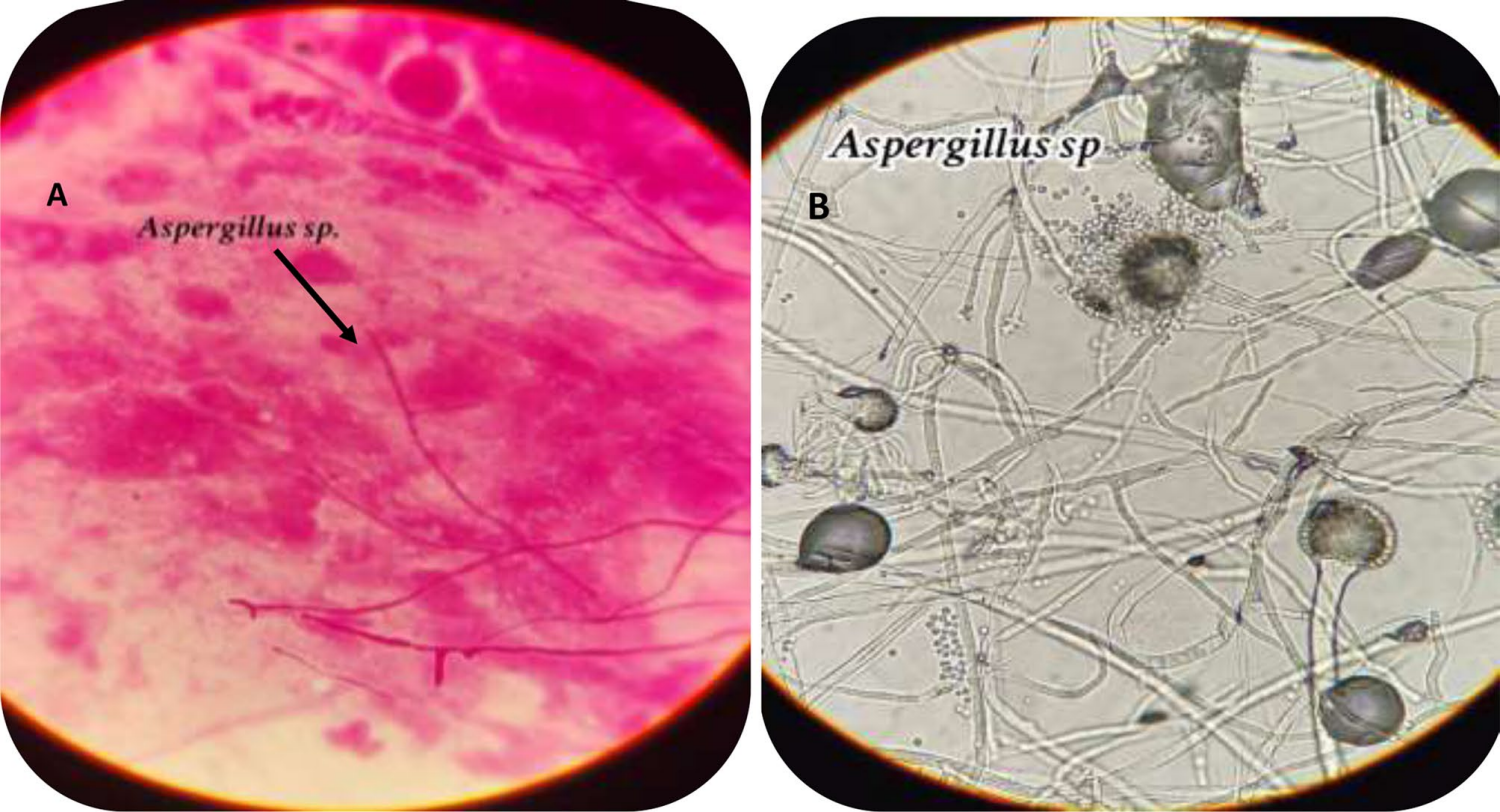
Microbiologic findings. (**A**) Gramstained corneal scraping shows septate, branching hyphae, consistent with a filamentous fungal pathogen (arrow). (**B**) After five days of incubation on Sabouraud dextrose agar, microscopic examination of the *Aspergillus* spp. colony growth revealed long, thread-like hyphae with a swollen, rounded vesicle at the tip at 400× magnification

On the first day, Gram stain revealed branching, Gram-positive septate, hyaline, fungal hyphae (Fig. 2A). Consequently, topical betamethasone was discontinued, and treatment was started with topical voriconazole 1% applied hourly, and levofloxacin 0.5% four times daily for prophylactic purposes. Because of significant stromal keratolysis and peripheral corneal–limbal involvement, oral voriconazole 200 mg twice daily was initiated, and early application of cyanoacrylate glue along with a single intrastromal voriconazole injection was performed. Natamycin 5% eye drops were not available in Iran. Fungal growth observed on Sabouraud dextrose agar showed morphological features consistent with *Aspergillus* spp. after five days; therefore, fungal PCR was not performed. Bacterial cultures and a herpes simplex virus (HSV) PCR test were negative, leading to discontinuation of oral acyclovir. (Fig. 2B).

After successful treatment, the patient was discharged without any complications, and topical voriconazole 1% was continued. Three months later, the glue was removed, and the infiltration responded well to treatment. A 5 × 5 mm superficial scar was detected in the inferonasal corneal–limbal area. (Fig. 1B). At six months of follow-up, BCVA improved to 0.2 LogMAR. Due to the presence of a peripheral corneal scar, penetrating keratoplasty was not performed.

### The dosage regimen for voriconazole

Fortified eye drops were prepared in the Department of Ocular Pharmacology at Farabi Eye Hospital, Tehran University of Medical Sciences. All steps were carried out under sterile conditions. For topical voriconazole 1%, 200 mg of voriconazole powder was dissolved in 19 mL of Ringer’s lactate solution. For the intrastromal injection, 50 mg (0.1 mL) of this solution was injected into the midstroma of the ulcer, away from the thinned area, using a 1 mL tuberculin syringe with a 30gauge needle, entering from a clear area of the cornea at an oblique angle.

## Discussion

A corneal ring infiltrate presents a diagnostic challenge, as it may occur in several infectious and noninfectious conditions [1]. In *Acanthamoeba* keratitis (AK), ring infiltrates typically appear early and are associated with severe pain disproportionate to clinical findings [3]. In bacterial keratitis, particularly *Pseudomonas* infection, a ring may develop rapidly as part of an immune response [2, 3]. In fungal keratitis, ring infiltrates are less common and tend to develop later in the disease course, often following progression or treatment, as seen in our case. Recognition of these distinctions is critical to avoid misdiagnosis and inappropriate therapy [4, 5]. The classic sterile immune ring, also known as the Wessely ring, is caused by a type III hypersensitivity reaction, the deposition of antigen-antibody complexes, activation of the complement system, and accumulation of polymorphonuclear neutrophils (PMNs) [7, 8]. This immunological ring is most commonly associated with *Acanthamoeba* and herpes simplex keratitis [5]. In a clinical series by Ross et al., ring infiltrates were reported in approximately 29.3% of AK cases, often observed in the early weeks of the disease course and reflecting an immune response to persistent protozoal antigens [9]. In herpes simplex keratitis, Meyers-Elliott et al. demonstrated immune rings resulting from stromal deposition of viral antigens and subsequent antigen–antibody complex formation, supporting a hypersensitivity mechanism [10].

Immune ring formation in FK has been documented in a limited number of case reports and small case series. Table 1. *Fusarium* spp. are the most common organisms, while *Aspergillus* spp. is less common [11, 12]. Although sterile immune ring in FK is generally measured as narrow in width, compared to those seen in AK and bacterial keratitis, there is no evidence suggesting that the morphology varies among different fungal genera [13]. Moreover, in FK, the immune ring develops more slowly and appears later, due to disease progression or after the initiation of antifungal therapy. This supports the hypothesis that the release of fungal antigens triggers a delayed host immune response, rather than indicating direct fungal extension [12–14]. Consistent with the previous study, in our case, *Aspergillus* spp., as an uncommon organism associated with immune ring formation, caused a narrow, width immune-like ring approximately three weeks after the onset of symptoms.

**Table 1:** Review for fungal keratitis with ring infiltration

Author (Year)	Fungal organism	Description of infiltration	Immune ring characteristics	Prior steroid use	Treatment	Surgical intervention	Outcome	Follow up duration	Final BCVA	References
Agarwal and Anand (2021)	Not specific only filaments on KOH ^1^	Central infiltration with surrounding ring infiltrate, initially suspected *Acanthamoeba*	4 mm ring infiltration	No	Natamycin 5%	No	Resolution infiltration in 2 weeks Infection healed	6 months	Not reported	[14]
Chaniyara, MH (2017)	*Aspergillus flavus*	Bilateral ring infiltrates with epithelial defect and surrounding corneal haze or edema, dry appearance	3mm wide ring infiltration in the right eye reaches the limbus and 2mm wide ring infiltration in the left eye	No	1.Natamycin 5% 2.Topical voriconazole 1% for 6 weeks	No	Right eye poor Left eye good and improve corneal clarity	6 months	Right eye 20/600 Left eye 20/20	[17]
Raghavan, A (2019)	Co-infection *Acanthamoeba* and fungi (*Fusarium*, *Aspergillus sp,*^*2*^)	Stromal edema, yellowish stromal infiltration, hyphate edges	Yellowish ring infiltrates with hyphate edges	No	1.PHMB^3^ 0.02%–0.06% or 2. Chlorhexidine 0.02–0.2% 3. Propamidine isethionate 0.1% 4.Natamycin 5% 5. Topical voriconazole 1%	No	Not reported	Not reported	Not reported	[4]
Zhao Y (2025)	*Phaeoisaria* *clematidis*	Deep central corneal ulcer with stromal infiltrate and irregular borders	Not reported	yes	1.Amphotericin B 0.5% 2.Oral ketoconazole 400 mg/day 3.Topical ofloxacin 4.Topical dexamethasone	T-PKP^4^	Ulcer healed and loukomatous opacity formed at 6 weeks	Not reported	Not reported	[18]
Atighehchian M (2024)	Co-infection *Acanthamoeba* and *Fusarium* sp.^2^	Progressive 7 × 7 corneal ulcer, mild stromal edema, satellite lesions, keratic precipitates, hypopyon	Grayishwhite ring -like stromal infiltration	No	1.Voriconazole 1% 2.Oral voriconazole 200 mg twice daily 3.PHMB^3^ 0.02%	T-PKP^4^	BCVA^5^ improved and no further complications reported at 6 months	6 months	1.0 LogMAR	[5]

1: KOH= Potassium Hydroxide, 2; Sp, = Species,3: PHMB=Polyhexamethylene biguanide,4: T-PKP=Therapeutic Penetrating keratoplasti,5: BCVA=Best corrected visual acuity, 6: LogMAR= Logarithm of the minimum angle of resolution

Using corticosteroids in the setting of an immune ring keratitis is challenging, as they may suppress local host defenses [15, 16]. If fungal pathogens are present, corticosteroids may promote fungal growth, worsen corneal infiltration and necrosis, and increase antigen release, thereby triggering a hypersensitivity response that can lead to immune ring formation later in the disease course [17, 18]. In the present case, due to the previous history of an old corneal scar, a coexisting fungal and viral infection could not be excluded initially. However, viral etiology was ruled out by PCR, and the favourable clinical response to antifungal therapy supports the diagnosis of FK. Prior topical betamethasone contributed to worsening stromal infiltration and may develop a narrow, delayed immune-like ring infiltrate.

In conclusion, fungal keratitis is less commonly associated with immune ring infiltrates, and *Aspergillus* spp. are relatively uncommon fungal pathogens associated with immune ring formation. Our case of *Aspergillus* spp. keratitis highlights this unusual association, as it presented with a delayed, narrow, immune-like ring. Although immune rings may appear late in fungal keratitis, topical corticosteroid therapy may have influenced ring formation in this case. This case highlights a rare and clinically important presentation of Aspergillus keratitis with delayed immune-like ring formation, initially misdiagnosed as herpetic keratitis and modified by prior corticosteroid use. The combination of these factors underscores the importance of microbiological confirmation before initiating steroid therapy in cases of ring infiltrates.

## Data Availability

The datasets used and/or analyzed during the current study available from the corresponding author on reasonable request.
